# Association between neighbourhood characteristics and antidepressant use at older ages: a register-based study of urban areas in three European countries

**DOI:** 10.1136/jech-2020-214276

**Published:** 2020-06-19

**Authors:** Lasse Tarkiainen, Heta Moustgaard, Kaarina Korhonen, J Mark Noordzij, Marielle A Beenackers, Frank J Van Lenthe, Bo Burstrom, Pekka Martikainen

**Affiliations:** 1 Population Research Unit, University of Helsinki Faculty of Social Sciences, Helsinki, Finland; 2 Helsinki Institute of Urban and Regional Studies, University of Helsinki, Helsinki, Finland; 3 Public Health, Erasmus Medical Center, Rotterdam, Netherlands; 4 Department of Human Geography and Spatial Planning, Utrecht University, Utrecht, Netherlands; 5 Department of Global Public Health, Karolinska Institutet, Stockholm, Sweden; 6 Max-Planck-Institute for Demographic Research, Rostock, Germany; 7 Department of Public Health Sciences, Stockholm University, Stockholm, Sweden

**Keywords:** Mortality, demography, social inequalities, depression, cohort studies, health services, geography, gis, mental health, health behaviour, epidemiology, health inequalities, neighborhood/place, public health, social epidemiology, access to hlth care, inequalities, socio-economic, ageing, registers, marital status

## Abstract

**Background:**

Research evidence on the association between neighbourhood characteristics and individual mental health at older ages is inconsistent, possibly due to heterogeneity in the measurement of mental-health outcomes, neighbourhood characteristics and confounders. Register-based data enabled us to avoid these problems in this longitudinal study on the associations between socioeconomic and physical neighbourhood characteristics and individual antidepressant use in three national contexts.

**Methods:**

We used register-based longitudinal data on the population aged 50+ from Turin (Italy), Stockholm (Sweden), and the nine largest cities in Finland linked to satellite-based land-cover data. This included individual-level information on sociodemographic factors and antidepressant use, and on neighbourhood socioeconomic characteristics, levels of urbanicity, green space and land-use mix (LUM). We assessed individual-level antidepressant use over 6 years in 2001–2017 using mixed-effects logistic regression.

**Results:**

A higher neighbourhood proportion of low-educated individuals predicted lower odds for antidepressant use in Turin and Stockholm when individual-level sociodemographic factors were controlled for. Urbanicity predicted increased antidepressant use in Stockholm (OR=1.02; 95% CI 1.01 to 1.03) together with more LUM (OR=1.03; 1.01–1.05) and population density (OR=1.08; 1.05–1.10). The two latter characteristics also predicted increased antidepressant use in the Finnish cities (OR=1.05; 1.02–1.08 and OR=1.14; 1.02–1.28, respectively). After accounting for all studied neighbourhood and individual characteristics of the residents, the neighbourhoods still varied by odds of antidepressant use.

**Conclusions:**

Overall, the associations of neighbourhood socioeconomic and physical characteristics with older people’s antidepressant use were small and inconsistent. However, we found modest evidence that dense physical urban environments predicted higher antidepressant use among older people in Stockholm and the Finnish cities.

## INTRODUCTION

Research on associations between neighbourhood characteristics and mental health is extensive, and has expanded in recent decades. The evidence base concerning such an association at older ages is, however, thin and inconclusive despite the ageing populations and still ongoing urbanisation of most industrialised countries. Older people may be particularly susceptible to neighbourhood factors,^1^ being less likely to commute and to spend time outside the neighbourhood. Those with functional limitations, in particular, are restricted to the immediate surroundings of the home. There are numerous mechanisms through which neighbourhood characteristics could affect mental health. Access to green space may reduce stress and be beneficial, for example, whereas a socioeconomically deprived area may be stressful and lack services, which may have harmful repercussions.^2^ Although some studies report no association between neighbourhood social characteristics and depressive scores, or changes in depressive scores,^3–^ ^5^ an association between neighbourhood disorder and an increase in depression status was found in one study, but only among the non-married elderly.^6^

The evidence from younger age groups implies poorer mental health in socioeconomically deprived neighbourhoods but it is unclear whether or not the association is causal.^2 7 8^ The majority of previous studies are cross-sectional in design, thereby undermining causal inference. Longitudinal studies have also shown inconsistent results. Studies with follow-ups of less than 5 years suggest independent associations between neighbourhood socioeconomic characteristics and depression. However, studies with at least 5 years of follow-up indicate that the association is attributable to compositional effects, that is, residents in deprived neighbourhoods had more often individual sociodemographic characteristics increasing the risk of depression, and accounting for these individual-level characteristics attenuated the observed area-level association.^8^ These studies assessed specific social or physical features of neighbourhoods, which are difficult to observe and measure directly and objectively, and therefore used neighbourhood socioeconomic indicators aggregated from the individual level as proxies for these features.

Several studies in recent years have explored more direct associations between mental health or psychological distress and the physical characteristics of urban environments instead of using neighbourhood characteristics aggregated over individuals.^9–^ ^12^ According to studies using register or satellite-based land-use data, green space in the neighbourhood is beneficial to mental health,^13 14^ although not all studies report an association.^10^ ^15–^ ^18^ Evidence regarding land-use mix (LUM) or walkability is also inconclusive.^10 12 19^ One study specifically focusing on older populations reported no association between changes in green space and mental health over time, despite observing a cross-sectional association.^18^

Overall, evidence on the association between urban neighbourhood characteristics and mental health at older ages is limited and mixed. However, comparative studies between countries are scarce although it is possible that the underlying mechanisms are specific to national contexts (eg, the general level of segregation, welfare and healthcare systems) or to factors that affect how residents interact with their urban surroundings (eg, climate and cultural practices regarding the use of public spaces). It has also been suggested that heterogeneity in the measurement of mental-health outcomes, neighbourhood characteristics and confounders explains the inconsistency of findings in different settings.^9^

The current study is the first to assess the association between urban neighbourhood characteristics and mental health at older ages using uniform measures of exposure, outcomes and confounders based on register, census and satellite data in three national contexts including Finland, Sweden and Italy. We used antidepressant prescriptions or purchases identified from administrative drug-prescription registers as a measure of mental health. Antidepressant purchases do not perfectly identify all individuals with depression,^20^ but it can be considered as a complementary method for assessing mental health, which is extremely difficult to measure on the population level. Few studies thus far have taken advantage of available register data that also cover depressed individuals who are likely not to respond to surveys. Only two previous studies used individual-level data on psychotropic medication as a proxy for mental-health status: Maguire and colleagues^21^ showed that physical residential segregation predicted more antidepressant use, and the results of Melis *et al* ^22^ indicate that urban density and accessibility by public transport are slightly protective against being prescribed antidepressant medication.

This study enhances understanding of the links between specific neighbourhood socioeconomic and physical characteristics and mental health at older ages. The specific aims were to assess: (1) whether neighbourhoods differ in individual-level antidepressant use and is there an association between socioeconomic and physical neighbourhood characteristics and antidepressant use; (2) whether such associations are attributable to the individual sociodemographic characteristics of the residents (compositional effects); and (3) whether the findings are consistent across the three countries.

## DATA AND METHODS

We used register-based data linking individual-level information on prescriptions for or purchases of antidepressants (as a proxy for mental health) and sociodemographic characteristics with area-level information on neighbourhood socioeconomic and physical characteristics. The analysis was limited to individuals aged 50+. The Italian data covered the population of the city of Turin in 2001. The Swedish data included all persons residing in the Stockholm urban area (Stockholm city and urban parts of 11 adjacent municipalities) in 2011. In Finland, in order to increase statistical power in the analyses, we included the nine largest cities of Helsinki, Espoo (including Kauniainen), Vantaa, Turku, Tampere, Oulu, Jyväskylä, Kuopio and Lahti. The physical characteristics of the neighbourhoods were based on European Urban Atlas satellite imaging data (UA).^23^ These data were aggregated to the relevant neighbourhood level using a Geographic Information System (QGIS). See table 1 for detailed description of the data sets.

**Table 1:** Datasets used in the study

	Turin	Stockholm	Finnish cities
Data source*	Census linked to prescription register	Administrative registers and Swedish prescribed drug register	Administrative registers and prescription register
Sample coverage	100%	100%	11%+80% oversample†
Baseline year	2001	2011	2003
Follow-up period	2002–2007	2012–2017	2004–2009
Urban Atlas data year	2006	2012	2012
Number of areas	92 statistical zones	233 basic areas	339 postal codes
Median area population (IQR)	6761 (2358–12 790)	4926 (2654–8748)	3785 (1413–6649)

*Register linkages were authorised by national statistical or data protection authorities in each country.

†The Finnish dataset was a nationally representative 11% random sample of all Finnish residents in 2003. To increase the statistical power for small-area analysis, Statistics Finland amended the data with an 80% oversample of persons who died in 2004–2007 (probability weights were used in the analysis to account for the sampling design).

### Antidepressant use

The binary outcome variable was defined as whether an individual had made at least one purchase of or had been prescribed antidepressants during the 6-year follow-up period after baseline. We included codes N06A in the Anatomical Therapeutic Chemical Classification, excluding tricyclic antidepressants (codes N06AA but not N06AA22), which are often used for non-psychiatric indications at older ages.^24^

### Neighbourhood-variables and individual-level confounders

The neighbourhood level was delineated according to the postal-code (zip-code) area or equivalent. As people are less likely to move residence in older ages, we considered them to be exposed to the same baseline neighbourhood across the follow-up. Neighbourhoods with a very low number (n<50) of residents were removed (2 in Turin, 9 in Stockholm and 14 in the Finnish cities). Indicators of neighbourhood socioeconomic characteristics were aggregated from individual-level register or census data at each baseline year, including the proportion of residents with a basic education, the proportion of households living in rented dwellings and the unemployment rate. The physical characteristics were derived from UA data: the proportion of green areas (forests and parks) in the total neighbourhood area, the proportion of continuous urban fabric (henceforth referred to as urbanicity) and population density (residents per square kilometre). LUM was indicated by an entropy index, which varies between zero (when the neighbourhood has only one use such as industrial, green or continuous/discontinuous urban fabric) and one (when all uses are evenly present). The index was calculated as:

$$LUM=-\sum_{i=1}^{k}\frac{P_{i}\times \ln(P_{i})}{\ln(k)},$$

where *k* is the number of land-use categories (which varies between neighbourhoods) and *p* is the proportion of use *i* of the area.^25^ All neighbourhood-level variables were used as continuous variables and scaled into categories of 10-percentage-points in order to have a meaningful interpretation of the coefficients in that they indicate change in the odds of the outcome per 10-percentage-point increase in exposure. Population density was scaled into 10 000 persons per square kilometre.

Individual-level confounders measured at baseline included sex, age, education (high (International Standard Classification of Education (ISCED) 2011 levels 5–8), intermediate (ISCED 3–4) and basic (ISCED 0–2)), economic activity (employed, unemployed, retired, other), marital status (married, never-married, divorced, widowed), housing tenure (owner, renter, other) and household composition (living alone, other).

### Statistical methods

We used two-level random intercept logistic regression models in which the individuals were nested in neighbourhoods. Due to national data protection regulations, the Finnish, Swedish and Italian data were modelled separately. We estimated the ORs and the robust SEs for having at least one antidepressant purchase by neighbourhood-level indicators of the socioeconomic and physical environment. We first estimated the unadjusted models for each neighbourhood indicator, and then we adjusted for neighbourhood compositional effect by including individual-level confounders in the models. The models of the Finnish data also included a city covariate to account for the differences among the 9 cities included in the analysis.

To assess the magnitude of how neighbourhoods generally differ from each other by antidepressant use in each city, and not only the associations of specific neighbourhood characteristics, we estimated median ORs (MOR) for (1) the empty model, (2) the model including all individual-level characteristics and (3) the model including all individual-level characteristics separately for each neighbourhood indicator. MOR describes the heterogeneity of neighbourhoods. It is the median of ORs between two individuals with identical covariates from two random areas with different antidepressant uses. In practice, the MOR shows the extent to which the individual odds of antidepressant use is predicted by residential area.^26 27^ We also estimated intraclass correlations (ICC) using the latent response formulation to assess the extent of clustering in antidepressant use across neighbourhoods.^28^ For descriptive statistics, we calculated incidence rates for years on antidepressants during the follow-up per 1000 person years, adjusted for age using 2013 European Standard Population. All the statistical analyses were conducted using STATA 16.

## RESULTS

The age-adjusted rates for antidepressant purchases were lowest in Turin and highest in the Finnish cities, with higher rates among women, the divorced and widowed and those living alone in all countries (table 2). In Turin, individuals with a basic education, the unemployed and renters had lower rates of antidepressant use than the more privileged groups whereas the opposite was true for Stockholm and the Finnish cities. The proportions of basic educated, renters and unemployed were lower in Stockholm than in the other cities while the proportion of basic educated was notably higher in Turin than elsewhere (table 3). Compared to the other countries, the neighbourhoods in Turin were generally more densely populated and urban, whereas the Finnish neighbourhoods had more green areas. The LUM varied similarly in all three countries.

**Table 2:** The proportions of persons at baseline and age-adjusted incidence rates for antidepressant use by individual characteristics in Turin, Stockholm and certain Finnish cities

	% of persons			Rate		
	Turin	Stockholm	Finnish cities	Turin	Stockholm	Finnish cities
Age (mean)	66.1	65.2	63.7			
Sex						
Male	43	46	42	58.2	69.2	84.1
Female	57	54	58	103.7	119.5	130
Education						
Basic	73	24	44	82.2	100.2	113
Intermediate	17	44	26	86.4	97.2	110.9
High	10	32	30	86.8	93.8	107.5
Marital status						
Never-married	8	18	12	79.7	99	109.4
Married	66	49	55	80.8	81	98.1
Divorced	20	22	19	95	118.7	134.1
Widowed	6	11	15	96.8	118.4	135.4
Household composition						
Living alone	26	56	32	92.9	114.1	135.1
Other	74	44	68	81.3	83.7	100.2
Economic activity						
Employed	21	47	38	71.7	68.7	75.4
Unemployed	2	1	5	56.8	74.9	106.8
Retired	52	45	53	104.8	156.8	160
Other	26	7	3	80.6	85.4	91.2
Housing tenure						
Owner	71	69	70	83.8	88.2	103.2
Renter	25	31	25	81.7	114.8	133.5
Other	4	1	6	88.1	150.6	119.1
N	347647	431361	94347			

**Table 3:** The medians (and IQRs) of neighbourhood characteristics and age-adjusted incidence rates for antidepressant purchases in Turin, Stockholm and certain Finnish cities

	Median (IQR)			Incidence rate below–above median*		
Area-level variables	Turin	Stockholm	Finnish cities	Turin	Stockholm	Finnish cities
% Basic education	54 (38–66)	14 (11–20)	29 (24–35)	89.1–80.4	96.9–97.1	112.5–109.3
% Unemployment	10 (8–13)	01 (01–03)	10 (07–13)	88.4–79.4	94.4–99.1	111.9–110.5
% Renters	29 (24–33)	22 (05–39)	40 (23–53)	83.9–83.1	89.8–101.8	102.7–114.4
% Green areas	6 (4–16)	21 (12–32)	28 (16–47)	84.2–82.9	97.8–95.7	113.1–107.8
% Urbanicity†	26 (6–48)	0 (0–0.001)	0 (0–2)	81.4–84.5	94.3–100.5	106.3–113.3
Land-use mix	74 (65–80)	77 (69–82)	72 (54–79)	83.3–83.7	91.1–101.1	106.9–113.2
Population density‡	0.79 (0.16–1.34)	0.26 (0.15–0.48)	0.10 (0.02–0.21)	81.1–84.3	88.3–102	101.2–113.9

*Rate is years on antidepressants per 1000 person years in all the neighbourhoods below and above the area-level variable median, adjusted for age using 2013 European Standard Population.

†Percentage of dense urban fabric, 69% of areas in Stockholm had zero per cent of dense urban fabric and therefore incidence rates for Stockholm are provided here for areas without and with dense urban fabric.

‡10 000 residents per km^2^.

**Figure 1:**
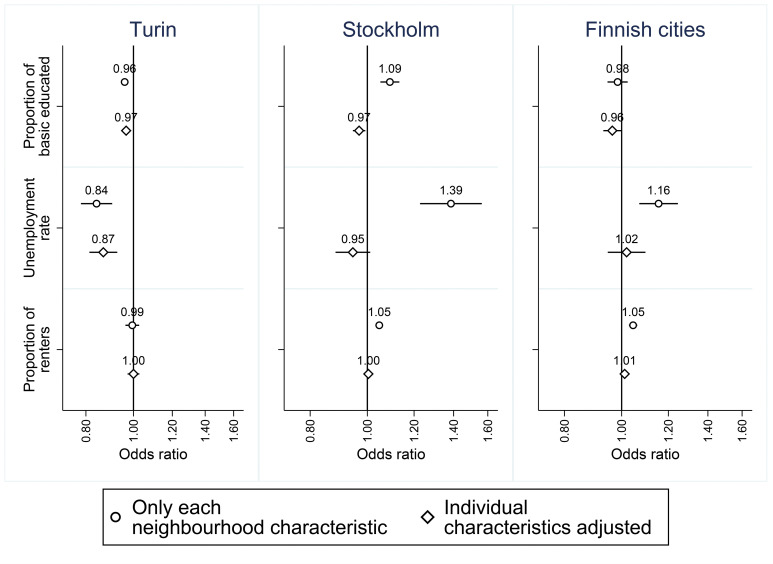
ORs and 95% CIs for socioeconomic neighbourhood-level characteristics in Turin, Stockholm and the nine largest Finnish cities from two models 1: Only each neighbourhood-level variable in the model, 2: each neighbourhood-level variable and all individual characteristics adjusted for (coefficients indicate change in the odds of the outcome per 10-percentage-point increase in exposure).

In the bivariate models, the neighbourhood unemployment rate and the proportion of renters predicted higher odds for antidepressant use in Stockholm and the Finnish cities, as well as the proportion of people with a basic education in Stockholm (figure 1). In the case of Turin, the proportion of people with a basic education and the unemployment rate were inversely associated with antidepressant use. Following adjustment for individual characteristics, reverse associations were observed for the proportion of residents with basic education in Stockholm (OR=0.97; 95% CI 0.95 to 0.99) and Turin (OR=0.97; 0.95–0.98), and for the unemployment rate in Turin (OR=0.87; 0.81–0.93).

**Figure 2:**
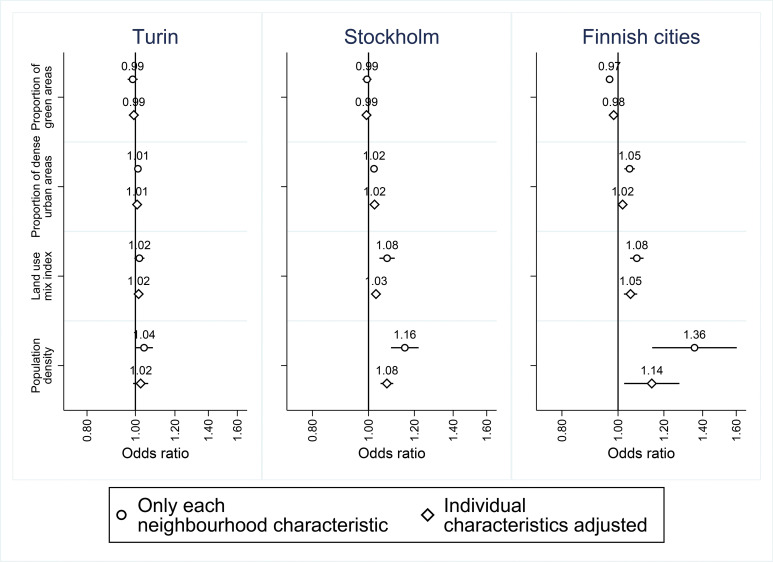
ORs and 95% CIs for physical neighbourhood-level characteristics in Turin, Stockholm and the nine largest Finnish cities from two models 1: Only each neighbourhood-level variable in the model, 2: each neighbourhood-level variable and all individual characteristics adjusted for (coefficients indicate change in the odds of the outcome per 10-percentage-point increase in exposure).

The patterns of bivariate associations between physical neighbourhood characteristics and antidepressant use were consistent across the countries, differing only slightly in magnitude. However, all 95% CIs included 1.00 in Turin (figure 2). A higher proportion of green areas predicted lower odds for antidepressant use only in the Finnish cities (OR=0.97; 0.95–0.98). Of the other neighbourhood characteristics, more densely populated, more urban and mixed urban structure predicted higher odds for antidepressant use. These associations were slightly attenuated following adjustment for individual characteristics, but remained in Stockholm and the Finnish cities in which population density was the strongest predictor (OR=1.08; 1.05–1.10 in Stockholm and OR=1.14; 1.02–1.28 in the Finnish cities).

The median OR, or MOR, between two individuals living in two random neighbourhoods with the differing neighbourhood-level risk of antidepressant use (ICC in parentheses), for the empty model was 1.11 (0.003) in Turin, 1.19 (0.010) in Stockholm and 1.17 (0.008) in the Finnish cities. When all the individual variables were included, MOR decreased to 1.08 (0.002) in Turin, 1.09 (0.003) in Stockholm and 1.14 (0.005) in the Finnish cities. Adding neighbourhood characteristics to the full model did not change these figures notably: the MORs were at most 0.02 lower (see online supplementary table S1).

10.1136/jech-2020-214276.supp1 Supplementary data

## DISCUSSION

We showed that a higher percentage of residents with only basic education in neighbourhood predicted a lower likelihood of antidepressant use in Turin and Stockholm, and that a higher population density and level of urbanicity as well as of mixed land-use led to increased antidepressant use in the Finnish cities and Stockholm. These associations were generally modest but were not attributable to compositional differences in the measured socio-demographic characteristics of residents between the neighbourhoods.

These findings are in line with results of previous studies reporting a higher prevalence of depressive disorders in urban areas, and an association between a higher population density and depression among older people.^29 30^ An association between diverse LUM and higher rates of depression has been reported among older men in Australia.^19^ However, other studies report no associations between urbanisation or population density and poor mental health, which may be attributable to the self-reported outcomes.^9 19^ The proportion of green areas in the neighbourhood was weakly inversely associated with antidepressant use in the bivariate model only in the Finnish cities. Although previous studies have attested to the protective effects of green areas, many of them concerned different mental-health outcomes and age ranges, or small sample sizes.^15^ The effect of green areas may differ along the life course and be particularly pronounced in childhood and adolescence.^14 31^ It has been assumed that the elderly generally spend more time in their residential area, but the weak to non-existent associations of green areas with antidepressant use we found is inconsistent with this view and warrants more detailed investigation on whether the quality of the green areas is more important than their total proportion.

The inverse associations we observed between antidepressant use and the proportion of neighbourhood inhabitants with a basic education, after accounting for individual-level characteristics, were counter to expectations. Instead of a high proportion of basic educated being protective against poor mental health, it is more likely that residents in these neighbourhoods are less likely to seek or receive antidepressant treatment for mental-health problems. Also at the individual level, following adjustment for all covariates, high education predicted higher odds of antidepressant use (online supplementary table S1). This implies that the association does not necessarily originate on the neighbourhood level, but that individuals with a low education are less likely to seek and receive treatment.

The differences in findings between Turin and Nordic urban areas, with stronger associations regarding physical characteristics in Nordic countries, may reflect well-established differences in family solidarity between the countries. Italy is characterised by strong family responsibility for older people while contact with elderly parents may be looser in the Nordic countries.^32^ Such differences may mean that older Finns and Swedes are more responsive to the characteristics of neighbourhoods than Italian older people, for whom the family context may be more relevant. In addition, Turin is altogether more densely populated and built (table 3). It is thus possible that the effects of urban characteristics on mental health vary at different parts of the distribution, with stronger effects seen in more loosely built environments.

The median ORs varied between 1.08 in Turin and 1.14 for the Finnish cities in the full model, indicating that individual-level characteristics did not fully explain all the neighbourhood differences in antidepressant use. This means that, on average, individuals in neighbourhoods with higher antidepressant use had 8–14% higher odds of using antidepressants compared to similar individuals in neighbourhoods with lower antidepressant use. However, given that the neighbourhood explained at most 0.5% of the total variation in antidepressant use (intraclass correlation), any association with antidepressant use appeared to be very modest overall. This implies that either the chosen area unit does not capture the effects of an urban environment on individual antidepressant use, or that the environment does not have a substantial impact. Thus, interventions aimed at improving the mental health of older individuals should be directed to contexts other than neighbourhoods (eg, families and communities). The very low level of clustering, as shown by the ICC, may also explain the inconclusiveness of previous results on this topic. As Merlo and colleagues^27^ point out, when clustering (or the general contextual effect) is small it is paradoxically easier to detect statistically significant coefficients for specific contextual effects; thus, even weak associations are reported. These weak-to-modest effects may be sensitive to study-specific national or urban characteristics and not generalisable or replicable in other studies.

### Strengths and limitations

Identifying mental-health outcomes from linked national medication registers enabled us to avoid common problems in surveys that relate to self-reported mental health, such as recall bias and preferential reporting,^33^ as well as the substantial non-response and loss to follow-up among socioeconomically disadvantaged and depressed population segments.^34^ In terms of neighbourhood-level measures, a clear strength of this study is the robust register-based information on both the socioeconomic and the physical characteristics, free from same-source bias and measured in a uniform and objective manner over several cities and national contexts. However, it is possible that the administrative-area units do not precisely coincide with residents’ perceptions of their neighbourhoods, which is likely to dilute the real effects of neighbourhood characteristics and result in conservative estimates. In addition, these data do not capture less severe forms of depression (not requiring pharmaceutical treatment), which may relate more strongly to neighbourhood characteristics.

The extent to which antidepressant prescriptions or purchases accurately measure an individual’s mental-health status depends on various factors. Neighbourhood and country differences in access to antidepressants (access to mental-healthcare, medication costs, reimbursements) may affect the willingness to seek treatment in a socioeconomically patterned manner, for example. Previous survey-based research evidence shows, contrary to our results, that the prevalence of depression among the elderly is higher in Southern European than in the Nordic countries.^35^ This suggests that despite the countries concerned having universal healthcare provision and heavily subsidised or completely free medications, we are possibly underestimating the prevalence of poor mental health in Turin. This may explain the different or absent associations observed in Turin. On the other hand, lower antidepressant use among those with a basic education, never-married and those living alone after all adjustments indicates the possible underestimation of mental-health problems among those with a low socioeconomic status in all studied countries (see online supplementary table S1). It should also be noted that not all individuals using antidepressants have mental-health problems. Antidepressants, tricyclics, in particular, are also prescribed for non-psychiatric indications including incontinence, sleep problems and pain.^23^ As we do not have information on diagnosis in the data, we aimed to increase the specificity of the measure by excluding tricyclic antidepressants from our analysis. The remaining misclassification increases measurement error in our outcome and possibly dilutes the estimated associations.^20^

Given that the onset of depression often occurs earlier in life than after the age of 50, the study subjects using antidepressants may have had mental-health problems before the baseline. This may have affected their choice of residence, as individuals with mental-health problems may be more likely to live in more densely populated neighbourhoods due to fewer financial resources. Moreover, specific personal characteristics (personality traits, genetic predispositions) may increase both the probability of residing in neighbourhoods with a high population density and being more susceptible to depression.^36 37^ Such patterned migration could have biased our results upwards. We therefore assessed the role of previous mental-health problems by excluding individuals with antidepressant use during the first 2 years of follow-up, and the results were very similar to our main analyses (results available upon request).

## CONCLUSION

We studied the association between mental-health status and neighbourhood characteristics in a uniform manner in different urban and national contexts. Overall, any associations of neighbourhood socioeconomic and physical characteristics with older people’s mental health were small and inconsistent. However, we found modest evidence of an association between a dense and mixed urban structure and higher levels of antidepressant use at older ages, particularly in the Stockholm area and the larger cities in Finland.

**What is already known on this subject**

- The physical and socioeconomic features of residential neighbourhoods may affect mental health.
- Studies on the association between neighbourhood characteristics and mental health at older ages have produced inconsistent findings, possibly due to heterogeneity in the measurement of mental-health outcomes, neighbourhood characteristics and confounders.

**What this study adds**

- Using uniform measures of exposure, outcome and confounders we found small and inconsistent associations between older people’s antidepressant use and most neighbourhood socioeconomic and physical characteristics in Turin, Stockholm and certain Finnish cities.
- We found modest evidence that a dense and mixed urban structure was associated with higher antidepressant use at older ages in Stockholm and in the Finnish cities.
