# Supplementary material for: Association between neighbourhood characteristics and antidepressant use at older ages: a register-based study of urban areas in three European countries
Source: J Epidemiol Community Health. 2020 Jun 19;75(5):426–32. doi: 10.1136/jech-2020-214276 (PMC8053343; doi:10.1136/jech-2020-214276)
Supplement: Supplementary data [file jech-2020-214276supp001.pdf]

**Supplementary Table S1. Odds ratios (OR) and median odds ratios (MOR) for physical and socioeconomic neighbourhood level characteristics in Turin, Stockholm and 10 largest cities in Finland from two models 1: Only each neighbourhood-level variable in the model 2: each neighbourhood-level variable and all individual characteristics adjusted for**

| | | Turin | | | | Stockholm | | | | Finnish cities | | | |
|---|---|---|---|---|---|---|---|---|---|---|---|---|---|
| | | Model 1: | | Model 2: | | Model 1: | | Model 2: | | Model 1: | | Model 2: | |
| **Neighbourhood characteristics** | | OR [95%CI] | MOR | OR [95%CI] | MOR | OR [95%CI] | MOR | OR [95%CI] | MOR | OR [95%CI] | MOR | OR [95%CI] | MOR |
| % Basic education | | 0.96 [0.95,0.97] | 1.08 | 0.97 [0.95,0.98] | 1.06 | 1.09 [1.05,1.13] | 1.18 | 0.97 [0.95,0.99] | 1.09 | 0.98 [0.95,1.02] | 1.17 | 0.96 [0.93,1.00] | 1.14 |
| % Unemployment | | 0.84 [0.78,0.90] | 1.09 | 0.87 [0.81,0.93] | 1.07 | 1.39 [1.23,1.56] | 1.18 | 0.95 [0.88,1.01] | 1.09 | 1.16 [1.07,1.25] | 1.16 | 1.02 [0.95,1.10] | 1.14 |
| % Renters | | 0.99 [0.96,1.03] | 1.11 | 1.00 [0.97,1.03] | 1.08 | 1.05 [1.04,1.06] | 1.15 | 1.00 [1.00,1.01] | 1.09 | 1.05 [1.03,1.06] | 1.15 | 1.01 [1.00,1.03] | 1.14 |
| % Green areas | | 0.99 [0.96,1.01] | 1.11 | 0.99 [0.97,1.02] | 1.08 | 0.99 [0.98,1.01] | 1.19 | 0.99 [0.98,1.00] | 1.09 | 0.97 [0.95,0.98] | 1.16 | 0.98 [0.97,1.00] | 1.13 |
| % Urbanicity * | | 1.01 [1.00,1.02] | 1.10 | 1.01 [1.00,1.02] | 1.08 | 1.02 [1.01,1.04] | 1.19 | 1.02 [1.01,1.03] | 1.09 | 1.05 [1.03,1.07] | 1.16 | 1.02 [1.00,1.04] | 1.14 |
| Land use mix | | 1.02 [1.00,1.04] | 1.11 | 1.02 [1.00,1.03] | 1.08 | 1.08 [1.04,1.11] | 1.18 | 1.03 [1.01,1.05] | 1.09 | 1.08 [1.05,1.11] | 1.15 | 1.05 [1.02,1.08] | 1.13 |
| Population density ** | | 1.04 [1.00,1.08] | 1.11 | 1.02 [0.99,1.06] | 1.08 | 1.16 [1.09,1.22] | 1.18 | 1.08 [1.05,1.10] | 1.08 | 1.36 [1.15,1.60] | 1.16 | 1.14 [1.02,1.28] | 1.13 |
| **Individual characteristics** | | | | | | | | | | | | | |
| Sex | Male | | | 1.00 | | | | 1.00 | | | | 1.00 | |
| | Female | | | 1.85 [1.80,1.90] | | | | 1.71 [1.67,1.74] | | | | 1.59 [1.53,1.66] | |
| Age | 50-54 | | | 1.00 | | | | 1.00 | | | | 1.00 | |
| | 55-59 | | | 1.01 [0.98,1.05] | | | | 0.92 [0.89,0.94] | | | | 0.78 [0.74,0.83] | |
| | 60-64 | | | 1.13 [1.09,1.17] | | | | 0.73 [0.71,0.76] | | | | 0.46 [0.42,0.51] | |
| | 65-69 | | | 1.34 [1.28,1.39] | | | | 0.50 [0.48,0.52] | | | | 0.37 [0.34,0.41] | |
| | 70-74 | | | 1.63 [1.56,1.70] | | | | 0.50 [0.47,0.52] | | | | 0.47 [0.43,0.52] | |
| | 75-79 | | | 1.80 [1.73,1.88] | | | | 0.60 [0.57,0.63] | | | | 0.54 [0.49,0.60] | |
| | 80-84 | | | 1.83 [1.74,1.93] | | | | 0.71 [0.68,0.75] | | | | 0.60 [0.53,0.67] | |
| | 85-89 | | | 1.39 [1.32,1.46] | | | | 0.75 [0.70,0.79] | | | | 0.54 [0.47,0.62] | |
| | 90+ | | | 0.88 [0.83,0.94] | | | | 0.54 [0.51,0.58] | | | | 0.34 [0.29,0.40] | |
| Education | Basic | | | 1.00 | | | | 1.00 | | | | 1.00 | |
| | Intermediate | | | 1.10 [1.07,1.12] | | | | 1.06 [1.03,1.08] | | | | 1.04 [0.99,1.09] | |
| | High | | | 1.12 [1.09,1.16] | | | | 1.07 [1.04,1.10] | | | | 1.08 [1.02,1.13] | |
| Marital status | Never-married | | | 1.00 | | | | 1.00 | | | | 1.00 | |
| | Married | | | 1.16 [1.10,1.22] | | | | 0.92 [0.89,0.95] | | | | 1.19 [1.11,1.28] | |
| | Divorced | | | 1.03 [0.98,1.07] | | | | 1.21 [1.18,1.24] | | | | 1.33 [1.25,1.42] | |
| | Widowed | | | 1.35 [1.29,1.42] | | | | 1.04 [1.01,1.07] | | | | 1.22 [1.14,1.32] | |
| Household composition | Living alone | | | 1.00 | | | | 1.00 | | | | 1.00 | |
| | Other | | | 1.03 [0.99,1.06] | | | | 1.04 [1.01,1.07] | | | | 1.18 [1.10,1.25] | |
| Economic activity | Employed | | | 1.00 | | | | 1.00 | | | | 1.00 | |
| | Unemployed | | | 1.20 [1.13,1.29] | | | | 1.48 [1.36,1.60] | | | | 1.27 [1.15,1.40] | |
| | Retired | | | 1.19 [1.15,1.24] | | | | 2.38 [2.30,2.45] | | | | 2.17 [2.01,2.34] | |
| | Other | | | 1.13 [1.09,1.17] | | | | 1.37 [1.31,1.42] | | | | 1.20 [1.07,1.35] | |
| Housing tenure | Owner | | | 1.00 | | | | 1.00 | | | | 1.00 | |
| | Renter | | | 0.98 [0.96,1.00] | | | | 1.20 [1.17,1.24] | | | | 1.26 [1.20,1.32] | |
| | Other | | | 0.98 [0.93,1.04] | | | | 1.48 [1.29,1.69] | | | | 1.01 [0.90,1.13] | |

* 10000 residents per $km^2$

** Percentage of dense urban fabric
